# Use of quality checks and processes across digital histopathology: an initial survey from the Bigpicture consortium

**DOI:** 10.1136/jcp-2024-210010

**Published:** 2025-07-11

**Authors:** Hayley Pye, David S Brettle, Caitríona Lyons, Fauve Versaevel, Erio Barale-Thomas, Kurt Zatloukal, Darren Treanor, on behalf of the Bigpicture consortium

**Affiliations:** 1 National Pathology Imaging Co-operative, Leeds Teaching Hospitals NHS Trust, Leeds, UK; 2 Deciphex Ltd, Dublin, Ireland; 3 A Johnson & Johnson Company, Janssen Pharmaceutica NV, Beerse, Belgium; 4 Institute of Pathology, Medical University of Graz, Graz, Austria; 5 Department of Clinical and Experimental Medicine, Linkopings Universitet, Linköping, Sweden

**Keywords:** Quality Assurance, Health Care, QUALITY CONTROL, Laboratory Manual

## Abstract

**Aims:**

In the end-to-end digital pathology workflow, variability can be introduced at each step, resulting in differences in the final image dataset. The effectiveness of quality control processes at each step of the workflow will impact the extent and relevance of this variability.

**Methods:**

To assess the maturity of whole slide imaging (WSI) quality processes for the whole digital pathology workflow, we conducted an online questionnaire across 19 digitally active members of the Bigpicture consortium.

**Results:**

A key finding was that a lower proportion of centres are implementing rigorous quality processes and checks processes at the post-scanning steps of the WSI workflow, such as ‘digital reporting and display’ (44%) and computational analysis (34%), when compared with pre-scanning steps such as ‘pre-staining’ (72%) and ‘staining’ (77%).

**Conclusions:**

This information allows us to identify priorities for quality improvement of the overall WSI workflow.

**WHAT IS ALREADY KNOWN ON THIS TOPIC**

- Quality control and assurance is important for limiting the variability that can be introduced at each step of the digital pathology workflow.

**WHAT THIS STUDY ADDS**

- A better description of the maturity and extent of digital pathology quality processes that are used across the digital pathology workflow in 19 clinical and non-clinical digital pathology centres across Europe.

**HOW THIS STUDY MIGHT AFFECT RESEARCH, PRACTICE OR POLICY**

- This information allows us to identify priorities for further quality improvement of the overall digital pathology workflow.

## Introduction

 Digital technologies are transforming the practice of histopathology. Advances in slide scanning and data storage hardware have revolutionised the field by enabling high-throughput whole slide imaging (WSI) of histopathological specimens. This has been rapidly followed by the acceleration of software solutions, including those employing computational and statistical analysis techniques, such as artificial intelligence (AI).^1^ The potential impact of computational pathology in its various forms across the entire pathway from basic and preclinical research to clinical diagnostics is substantial.^2 3^ There is a key role for pathologists as end-users of digital pathology workflows to help manage this. However, several challenges must be addressed regarding large-scale development, validation and adoption before this potential can be fully realised.^4 5^

**Figure 1:**
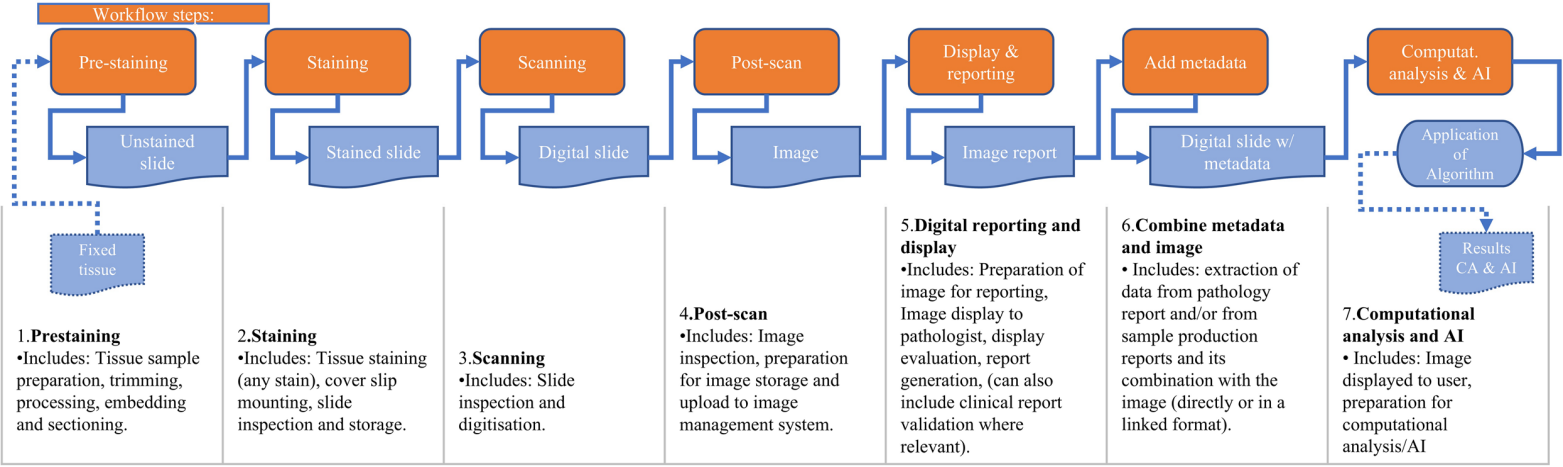
The seven different steps of WSI production. The proposed designation of a workflow for WSI production is divided into seven discrete steps. The seven steps of WSI production are shown in the orange boxes and are entitled: (1) pre staining, (2) staining, (3) scanning, (4) post-scan, (5) digital reporting and display, (6) reporting of metadata and (7) computational analysis and AI. This workflow was provided to respondents alongside the survey asked over the seven steps. (AI, artificial intelligence; CA, computational analysis; WSI, whole slide imaging.)

There are several steps in the WSI workflow from tissue preparation and staining through to digitisation and analytics (figure 1). Variability can be introduced and compounded at each step, resulting in a variable dataset. This variability could subsequently affect the dataset’s utility for image processing and AI applications.^6^,^8^ Developers of AI systems often address the issue of image variation by increasing the dataset size and/or introducing artificial variation or data augmentation, with the aim of allowing deep learning to mitigate the impact on system performance.^9^,^11^ However, the limits of these approaches are not yet fully understood. At a minimum, the variation in image generation should be better described and measured to allow for improved assessment of its impact. It is also important to note that if data are compromised or missing during acquisition, it cannot be rectified afterwards, which could affect the utility of the dataset, either for diagnosis or for the development and portability of computational methods.

The availability of quality control (QC) approaches for the production of pathology slides is understandably more established, guidance often being provided by the myriad of public and/or regulatory bodies across countries as well as across, clinical, non-clinical and veterinary pathology and good laboratory practice generally. The introduction of WSI scanning and computational processes to this workflow, however, provides unique challenges. These additions not only require understanding, development and validation of brand new QC processes, but review and re-validation of the appropriateness of those that are already in place for the earlier stages. This is because the end point of the workflow has changed completely in its nature and in many cases also its end use.

The Bigpicture project (https://Bigpicture.eu/) is a pre-competitive public-private partnership funded under the Innovative Health Initiative programme. It is aimed at establishing a comprehensive repository of 3 million digital pathology whole slide images, with a strong focus on the quality of both the images and the associated metadata. The project’s consortium comprises a collaborative community, including clinical and non-clinical pathologists, researchers, AI developers, patients and industry stakeholders. A key objective of the Bigpicture project is to understand and disseminate information on WSI quality and to use tools for measuring and improving this quality. To achieve this, a foundational understanding of the current WSI quality landscape is essential. The data presented represent an initial effort towards establishing this baseline.

## Materials and methods

For this cross-sectional study, the survey questions were developed by members of the Quality Coordination Centre at National Pathology Imaging Cooperative (https://npic.ac.uk/quality-coordination-centre/), with review and input from members of the Bigpicture Task Force Quality Coordination Centre (https://Bigpicture.eu/). An earlier version of the survey underwent anonymous pre-testing with a smaller group of Bigpicture quality coordinators (n=9) during a workshop held the previous year. The final survey was sent to 23 Bigpicture quality coordinators, all partners to the Bigpicture consortium. Non-responders were contacted via targeted email up to four times during the open submission period. Completed surveys were received from 19 centres (83%).

Data collection was conducted pseudo-anonymously to prevent multiple participations, and used the ‘Advantage Plan Survey Monkey Platform’ by Momentive.ai Inc (https://www.surveymonkey.com/). The estimated time to complete the survey was 10 min. While the survey did not require specific preparation or training, some submission guidance was provided with the survey, and this guidance is included in the online supplemental file 1, along with a copy of the survey. Respondent pseudo-anonymity and hence confidentiality were maintained, with identities known only to a limited number of team members who did not participate in the survey themselves. Access to the results was similarly restricted, ensuring that respondents could not view each other’s responses. On export from the platform, raw data onward were only used in its pseudo-anonymised form. The link to the identifying names of individuals or centres was stored in a single place which was secure and had both limited access and password protection. Only anonymised summary data were shared back to respondents and included in any subsequent reports.

All questions were structured across the seven discrete steps of the WSI production workflow (termed ‘stages’ in the original survey, but ‘steps’ here for clarity):

1. Pre-staining.
2. Staining.
3. Scanning.
4. Post-scan.
5. Digital reporting and display.
6. Combine metadata and image.
7. Computational analysis and AI.

**Figure 2:**
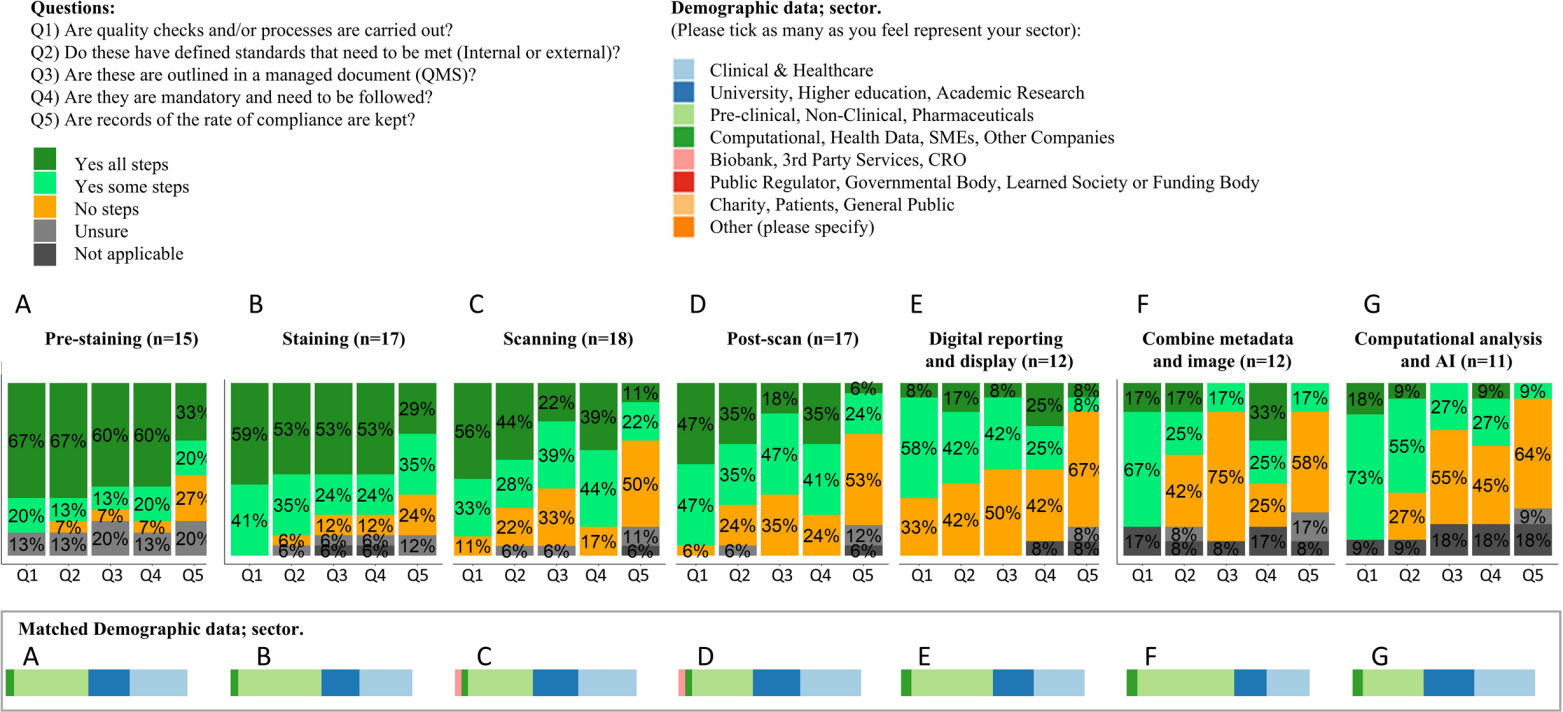
Landscape data showing the distribution of answers to five questions about quality management asked over the seven different steps of WSI production. The seven steps of WSI production are further detailed in figure 1 and are entitled: (**A**) pre staining, (**B**) staining, (**C**) scanning, (**D**) post-scan, (**E**) digital reporting and display, (**F**) combine metadata and image (**G**) computational analysis and AI. For each step, the same five questions were asked: (1) Are quality checks and/or processes carried out?, (2) Do these have defined standards that need to be met (internal or external)?, (3) Are these outlined in a managed document (QMS)?, (4) Are they mandatory and need to be followed? and (5) Are records of the rate of compliance kept?. The only available answers for all questions were ‘Yes all steps’, ‘Yes some steps’, ‘No’, ‘Unsure’ or ‘Not applicable’. Contributors were allowed to opt out of steps where they had no expertise on or experience with. Demographic data were collected alongside the survey data and are shown in the bottom half of the figure for the contributors who answered each step. (QMS, Quality Management System; WSI, whole slide imaging; CRO, Clinical Research Organization; SME, Small and Medium-sized Enterprise.)

Reporting of metadata associated with an image is often overlooked, but in this new era of computational analysis, having good quality and appropriate metadata will be crucial for the successful implementation of digital images within the range of different computational pathways/AI models that may be available to a user in future. Hence, we have included this as a unique step. These steps are further detailed in figure 1. This figure was provided to the respondents to facilitate survey completion. It was anticipated that some consortium members might not engage in all steps of the WSI workflow, as such an opt-out question was included at the beginning of each step: ‘Are you able to answer questions on this step of WSI production?’ The number of respondents per step is displayed in figure 2. The distribution of steps for which respondents provided data is as follows:

- 1 step (n=0).
- 2 steps (n=3).
- 3 steps (n=1).
- 4 steps (n=1).
- 5 steps (n=3).
- 6 steps (n=3).
- 7 steps (n=8).

Respondents who did not opt out of a step were required to answer every question in that step. To ensure completeness, the survey did not permit incomplete data submission within a step, classifying any missing data as MNAR (missing not at random), with rates clearly indicated in the figures and tabulated data provided in this manuscript. A free-text comment box was included for additional input. All responses were coded as categorical data at the respondent level.

Responses for all questions were restricted to:

- Yes, at all steps.
- Yes, some steps.
- No.
- Unsure.
- Not applicable.

For each of the seven steps, five questions were posed regarding quality checks and/or processes:

(Q1) Are quality checks and/or processes carried out?

(Q2) Do these checks have defined standards (internal or external)?

(Q3) Are these standards outlined in a managed document or QMS?

(Q4) Are the standards mandatory and required to be followed?

(Q5) Are records of compliance rates maintained?

Note, a ‘managed document’ refers to one which is version controlled and periodically reviewed. These are often used with a ‘QMS’ which stands for a ‘Quality Management System’. A QMS is a collection of defined processes, procedures and responsibilities that are carried out on a system (in this case, the digital pathology workflow) to help it better meet its objectives alongside improving effectiveness and efficiency.

Two additional questions were asked for each of the seven steps to capture respondents’ opinions on the following two statements:

(Q6) How much does the variation at each step concern you in digital histopathology?

(Q7) How much do you believe image/data processing can eliminate the need for improved quality checks at each step?

Responses to these questions were voluntary and were coded as categorical data using a 5-point Likert scale of:

- A great deal/Almost entirely.
- A little bit,
- I am not sure.
- Not very much.
- I don’t think it is a problem/I don’t think it can.

**Figure 3:**
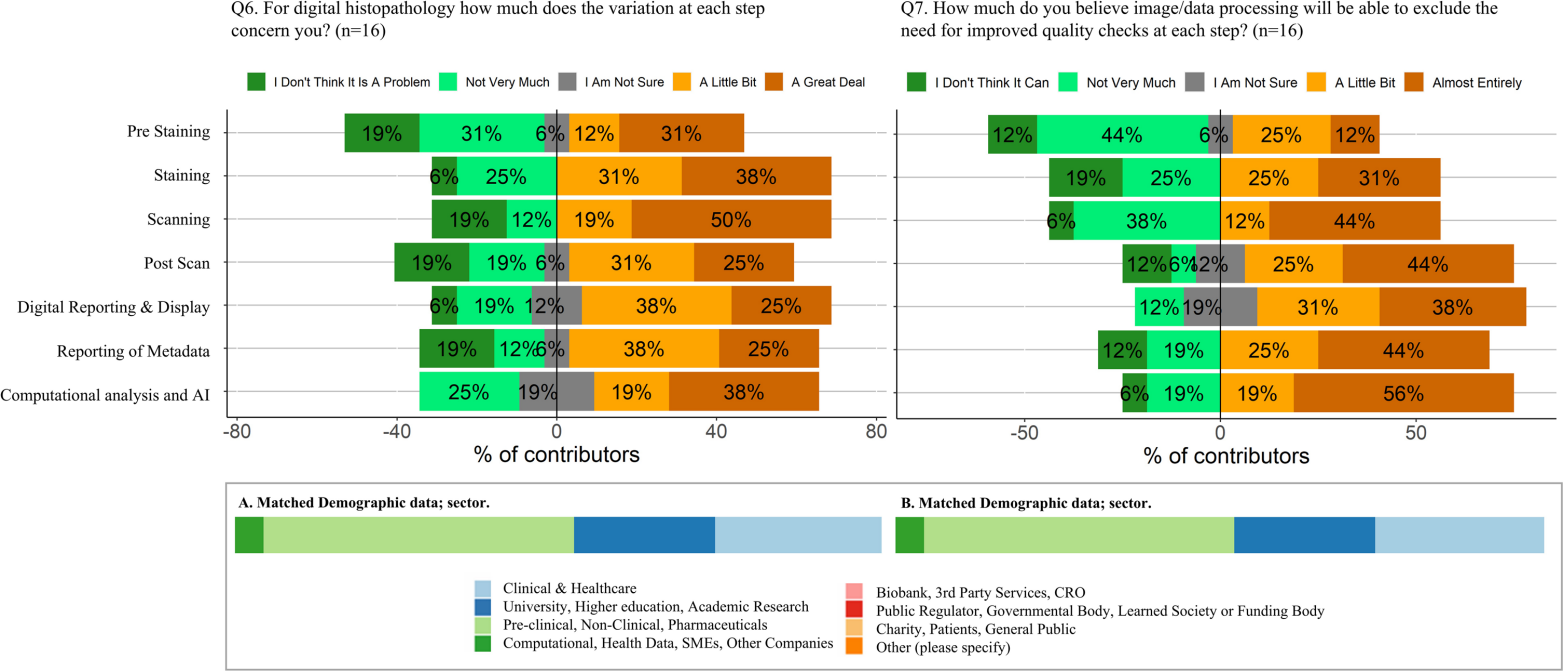
Opinion data showing the percentage of respondents that answered two questions about variation within digital histopathology. Respondents were asked to provide their opinion over a 5-point Likert scale to two questions (**Q6**) “For digital histopathology, how much does the variation at each step concern you?” and (**Q7**) “How much do you believe image/data processing will be able to exclude the need for improved quality checks at each step?” The percentage of respondents for each question is shown as coloured sections of a bar. The dark and light green bars reflect answers on one side of the scale (low concern or low confidence), the grey bar is a neutral answer and the dark and light orange bars reflect answers on the other end of the scale (high concern or high confidence). Contributors were allowed to opt out of each question if they wanted. Demographic data were collected alongside the questionnaire data and are shown in the bottom half of the figure for the contributors who answered each question. (AI, artificial intelligence; CRO, Clinical Research Organization; SME, Small and Medium-sized Enterprise.)

The number of respondents who provided a score for each statement is shown in figure 3.

Data were also collected on the self-reported demographics of the respondents. Respondents could choose multiple categories representing their workflow from the following list:

1. Clinical & Healthcare.
2. University, higher education, academic research.
3. Preclinical, non-clinical, pharmaceuticals.
4. Computational, health data, Small and Medium-sized Enterprises (SMEs), other companies.
5. Biobank, third-party services, Clinical Research Organization).
6. Public regulator, governmental body, learnt society or funding body.
7. Charity, patients, general public.
8. Other.

The sectors represented by the data are described in the results and shown alongside each step of the survey results in figures2  3, as well as in online supplemental file 1.

Data manipulation was carried out in RStudio V.4.2.1 (on 23 June 2022) running R V.4.1.2 (The R Foundation for Statistical Computing). Using the following packages: base, dplyr, ggpubr, ggplot2, gtsummary, gt, readr, reshape2, tidyr, ggrepel.^12^,^21^

The 'consensus-based checklist for reporting of survey studies' (CROSS) guidelines^22^ were reviewed in the reporting of this study. The checklist is available in online supplemental file 1.

### Patient and public involvement

No patients involved.

## Results

The results from the survey are presented in figure 2.

The results indicate that at least 65% of laboratories implement quality checks and/or processes at some or all steps of the workflow, with this figure rising to 90% for the ‘staining’, ‘post-scan’ and ‘computational analysis and AI’ steps. The quality of these checks is elaborated below. The step with the lowest reported percentage of quality checks and/or processes was ‘digital reporting and display.’ The proportion of laboratories reporting a complete absence of quality checks and/or processes was limited to the steps of ‘scanning’ (11%), ‘post-scan’ (5.9%) and ‘digital reporting and display’ (33%).

For the pre-scanning steps of ‘pre-staining’ and ‘staining’, an average of 72% and 77% of centres, respectively, reported that these checks had defined standards, were mandatory, had records of compliance kept, and were documented in a managed document for some or all steps. For the ‘scanning’ and ‘post-scan’ steps, this percentage dropped to 62% and 60%, respectively. It decreased further for the final three steps: ‘digital reporting and display’ (44%), ‘reporting of metadata’ (34%) and ‘computational analysis and AI’ (34%).

Demographic data collected alongside the survey data indicated similar demographics across each step, despite allowing step-specific opt-outs. The average demographic proportions across all steps were:

- 30% ‘Clinical & Healthcare’.
- 23% ‘University/Higher Education or Academic Research’.
- 40% ‘Pre-clinical or Non-Clinical Pharmaceuticals’.
- 5% ‘Computational/Health Data/SMEs or Other Companies’.
- 1% ‘Biobank/Third-Party Services or CRO’.

Respondents’ opinions are detailed in figure 3. The presented data suggest polarised opinions, with an average of 60% of respondents expressing concern about the variation introduced at all steps of digital histopathology workflow (either a great deal or a little bit), while 30% were not very concerned or not concerned at all. Only 7% were unsure. This pattern was similar in responses to the question about the extent to which image/data processing could obviate the need for improved quality checks at each step: 62% believed it could to some extent or almost entirely, 33% believed it would not do so or not very much and 5% were unsure.

## Discussion

The variability introduced across the WSI workflow has been recognised as a significant issue for digital pathology. This was highlighted in the 2019 recommendations from the Digital Pathology Association, which described ‘data variability’ and ‘the lack of standards’ as hurdles to implementation.^23^ Similarly, the 2021 best practice recommendations from the European Society of Digital and Integrative Pathology noted that ‘quality control (QC) practices in pathology laboratories across Europe were inconsistent’ and suggested a series of checkpoints to improve performance.^24^ Additionally, a report in 2021 outlining factors needing to be addressed to close the ‘translation gap’ for AI applications in digital pathology emphasised that ‘variability in tissue acquisition and histopathology slide preparation could impact the performance of downstream image analysis tools’ and that ‘this variability is best accounted for during model development and validation before widespread adoption’.^25^ This was addressed by the recently published ISO standard 20166-4:2021 ‘Molecular in vitro diagnostic examinations—Specifications for preexamination processes for formalin-fixed and paraffin-embedded (FFPE) tissue—Part 4: In situ detection techniques’,^26^ which specially refer to the increased quality requirements in the context of digital pathology and computational image analysis.

Previous surveys among experts have also documented this issue. A 2021 report by the National Physics Laboratory identified ‘an urgent need for… data and metadata standards, interoperability, QC measures and equipment calibration…’. The report also highlighted key gaps and priority issues aligning with those found in our work and showed that 68% of respondents thought that quality analysis (QA) in the ‘display’, ‘scanner’ and ‘sample’ were a medium or high priority for the near future.^27 28^ Another survey by Smith *et al* in Denmark ranked the statement ‘digital images have unsatisfactory quality’ as a top challenge in digital pathology.^29^ A survey by Pinto *et al* across Asia and Europe revealed a wide range of opinions on whether ‘digital pathology implementation improved the technical quality of slides’, likely reflecting variations in their WSI workflows.^30^

The aim of this study was to establish a baseline understanding of the maturity of quality processes within the WSI workflow among centres across the Bigpicture consortium, who may contribute to the Bigpicture dataset. The data presented here are the first step towards establishing that baseline but also providing an overview of the current landscape across various European laboratories.

Our data highlights the range of quality checks and processes across different steps of the WSI production workflow and identifies key gaps suitable for further investigation. For example, ‘digital reporting and display’ had the highest proportion of centres reporting no quality checks or processes. Despite a high proportion of centres carrying out some quality checks and processes for computational analysis and AI, these were less likely to be of ‘high quality’—that is, outlined in a managed document, mandatory or with recorded compliance. It was outside the scope of this survey, but it would also be worth investigating if the quality checks described here were manual or automated. In some cases, manual checks that are very tedious and time-consuming could be replaced by computational or computer-supported processes, for example, assessment of digital slide quality and the absence of artefacts. It is also worth considering that the extent and type of QC checks needed for computational analysis and AI will likely be different from those needed for quantitative digital assessment by a pathologist. There was a general trend of fewer and lower-quality processes and checks in the later steps of the WSI workflow, potentially due to a lack of available QA/QC tools or standards and guidelines in these key elements. This indicates a need for both novel QA/QC processes and education in these steps.

Variability tends to propagate throughout a workflow due to incremental and compounded errors, ending in the archived image. Clearly understanding where quality can be improved across the workflow is essential for enhancing the consistency of any archive.

Our presented opinion data, which was collected concurrently, asked if the variation introduced across the workflow was a concern. Results indicated a polarised group, with the majority expressing concern.

Data like ours, alongside other research showing the effects of variation on relevant endpoints, will help identify and prioritise the importance of addressing workflow variability, bringing the field closer to consensus and focusing on new or improved QA/QC tools. Our data suggest a focus on the last three stages that include digital reporting, display, metadata and computational analysis is the priority, with more work also needed around the point of scanning the slide. These areas should be targeted for the invention of both novel QA/QC processes and education in what can already be done at these steps to reduce the variability of a dataset.

Limitations of this work are: the relatively small sample size which might affect the generalisability of the findings. The fact that respondents were all highly experienced digital pathology leaders involved in the Bigpicture project shows selection bias. The study was not hypothesis-driven; therefore, it is only descriptive. And because all data were provided voluntarily and pseudo-anonymously, means where there are slight differences in contributors across the stages, sampling differences could influence the comparisons. Demographic data was collected to try and control for this last limitation. For future work, we would also add further clarification within the workflow diagram that AI or other computational analysis could be carried out at any step and not just within the final step termed ‘Computational analysis and AI’ (eg, in the scanning step an AI tissue detector could be used by the scanner). This is because this did cause a little confusion. For clarity, the final step only refers to the application of computational analysis and AI on a final dataset that is, i.e. for diagnosis. One final limitation is that ‘Computational/Health Data/SMEs’ was a demographic category in this study, but it represented only a small proportion of the respondents. It will be important to understand better this group’s perspective and experience as we move forward as a field.

The future expectation that managing variation that is casued by change in a digital pathology workflow will fall to the laboratoty itself, rather than by clinical or preclinical validation, are reflected in the updated validation guidelines from the College of American Pathologists. The 2013 statement says: ‘A completed validation study should provide a means to demonstrate that the WSI system validated can be used for the intended diagnostic purpose. However, whenever there is a significant change to the WSI system (eg, completely new type of scanner is used, major hardware or software upgrade) that may potentially affect the interpretation of digital slides, the validation process should be repeated with these new changes incorporated in the WSI system to demonstrate that it can still be used as intended.’ In the 2022 guideline, this was changed to ‘Laboratories should have procedures in place to address changes to the WSI system that could impact clinical results’.^31 32^

Following on from the work described in this study, we intend to carry out a piece of work describing current QC processes relevant to each stage in more detail. Ideally, this will also include consensus for which processes are thought to be most important at each stage.

The Bigpicture Task Force Quality Coordination Centre aims to coalesce the collective consortium expertise to quantify, understand and minimise variations in the WSI workflow. The data presented here will guide future work towards improved quality, fostering a culture of quality, and sharing data and guidelines on WSI quality with the broader community. We believe it is imperative for the wider community to produce the standardised, high-quality digital images for optimal patient outcomes, and a key objective will be creating and coordinating consensus and consistency in QCs and assurance processes to achieve this.

## Supplementary material

10.1136/jcp-2024-210010 online supplemental file 1

## Data Availability

All data relevant to the study are included in the article or uploaded as supplementary information.
