# Supplementary material for: Use of quality checks and processes across digital histopathology: an initial survey from the Bigpicture consortium
Source: J Clin Pathol. 2025 Jul 11;78(10):e210010. doi: 10.1136/jcp-2024-210010 (PMC12505092; doi:10.1136/jcp-2024-210010)
Supplement: online supplemental file 1 [file jcp-78-10-s001.pdf]

Supplementary Tabulated Data for Pye et al "**The use of quality checks and processes across digital histopathology: an initial survey from the Bigpicture consortium.**" **Page 1/3**

	Pre Staining, N = 19	Staining, N = 19	Scanning, N = 19	Post Scan, N = 19	Digital Reporting and Display, N = 19	Reporting of Metadata, N = 19	Computational Analysis and AI, N = 19
	**step QA).**	**step QB).**	**step QC).**	**step QD).**	**step QE).**	**step QF).**	**step QG).**
**Q1. Are Quality checks and/or processes are carried out**							
Yes All	10 (67%)	10 (59%)	10 (56%)	8 (47%)	1 (8.3%)	2 (17%)	2 (18%)
Yes Some	3 (20%)	7 (41%)	6 (33%)	8 (47%)	7 (58%)	8 (67%)	8 (73%)
No	0 (0%)	0 (0%)	2 (11%)	1 (5.9%)	4 (33%)	0 (0%)	0 (0%)
Unsure	2 (13%)	0 (0%)	0 (0%)	0 (0%)	0 (0%)	0 (0%)	0 (0%)
Not Applicable	0 (0%)	0 (0%)	0 (0%)	0 (0%)	0 (0%)	2 (17%)	1 (9.1%)
Opt Out	4	2	1	2	7	7	8
**Q2. Do these have defined standards that need to be met (Internal or external)**							
Yes All	10 (67%)	9 (53%)	8 (44%)	6 (35%)	2 (17%)	2 (17%)	1 (9.1%)
Yes Some	2 (13%)	6 (35%)	5 (28%)	6 (35%)	5 (42%)	3 (25%)	6 (55%)
No	1 (6.7%)	1 (5.9%)	4 (22%)	4 (24%)	5 (42%)	5 (42%)	3 (27%)
Unsure	2 (13%)	1 (5.9%)	1 (5.6%)	1 (5.9%)	0 (0%)	1 (8.3%)	0 (0%)
Not Applicable	0 (0%)	0 (0%)	0 (0%)	0 (0%)	0 (0%)	1 (8.3%)	1 (9.1%)
Opt Out	4	2	1	2	7	7	8
**Q3. Are these are outlined in a managed document or QMS**							
Yes All	9 (60%)	9 (53%)	4 (22%)	3 (18%)	1 (8.3%)	0 (0%)	0 (0%)
Yes Some	2 (13%)	4 (24%)	7 (39%)	8 (47%)	5 (42%)	2 (17%)	3 (27%)
No	1 (6.7%)	2 (12%)	6 (33%)	6 (35%)	6 (50%)	9 (75%)	6 (55%)
Unsure	3 (20%)	1 (5.9%)	1 (5.6%)	0 (0%)	0 (0%)	0 (0%)	0 (0%)
Not Applicable	0 (0%)	1 (5.9%)	0 (0%)	0 (0%)	0 (0%)	1 (8.3%)	2 (18%)
Opt Out	4	2	1	2	7	7	8
**Q4. Are they are mandatory and need to be followed**							
Yes All	9 (60%)	9 (53%)	7 (39%)	6 (35%)	3 (25%)	4 (33%)	1 (9.1%)
Yes Some	3 (20%)	4 (24%)	8 (44%)	7 (41%)	3 (25%)	3 (25%)	3 (27%)
No	1 (6.7%)	2 (12%)	3 (17%)	4 (24%)	5 (42%)	3 (25%)	5 (45%)
Unsure	2 (13%)	1 (5.9%)	0 (0%)	0 (0%)	0 (0%)	0 (0%)	0 (0%)
Not Applicable	0 (0%)	1 (5.9%)	0 (0%)	0 (0%)	1 (8.3%)	2 (17%)	2 (18%)
Opt Out	4	2	1	2	7	7	8
**Q5. Are records of the rate of compliance are kept**							
Yes All	5 (33%)	5 (29%)	2 (11%)	1 (5.9%)	1 (8.3%)	0 (0%)	0 (0%)
Yes Some	3 (20%)	6 (35%)	4 (22%)	4 (24%)	1 (8.3%)	2 (17%)	1 (9.1%)
No	4 (27%)	4 (24%)	9 (50%)	9 (53%)	8 (67%)	7 (58%)	7 (64%)
Unsure	3 (20%)	2 (12%)	2 (11%)	2 (12%)	1 (8.3%)	2 (17%)	1 (9.1%)
Not Applicable	0 (0%)	0 (0%)	1 (5.6%)	1 (5.9%)	1 (8.3%)	1 (8.3%)	2 (18%)
Opt Out	4	2	1	2	7	7	8

**Number of steps completed (out of 7)**	
Minimum	*2*
Mean	*5.3*
Maximum	*7*
0 steps completed	0
1 steps completed	0
2 steps completed	3
3 steps completed	1
4 steps completed	1
5 steps completed	3
6 steps completed	3
7 steps completed	8

Demographic	Pre Staining (n=22) step QA).	Staining (n=24) step QB).	Scanning (n=28) step QC).	Post Scan (n=27) step QD).	Digital Reporting and Display (n=18) step QE).	Reporting of Metadata (n=17) step QF).	Computational Analysis and AI (n=18) step QG).
Clinical & Healthcare	7 (32%)	7 (29%)	9 (32%)	9 (33%)	5 (28%)	4 (24%)	6 (33%)
University, Higher education, Academic Research	5 (23%)	5 (21%)	7 (25%)	7 (26%)	4 (22%)	3 (18%)	5 (28%)
Pre-clinical, Non-Clinical, Pharmaceuticals	9 (41%)	11 (46%)	10 (36%)	9 (33%)	8 (44%)	9 (53%)	6 (33%)
Computational, Health Data, SMEs, Other Companies	1 (4.5%)	1 (4.2%)	1 (3.6%)	1 (3.7%)	1 (5.6%)	1 (5.9%)	1 (5.6%)
Biobank, 3rd Party Services, CRO	0 (0%)	0 (0%)	1 (3.6%)	1 (3.7%)	0 (0%)	0 (0%)	0 (0%)
Public Regulator, Governmental Body, Learned Society or Funding Body	0 (0%)	0 (0%)	0 (0%)	0 (0%)	0 (0%)	0 (0%)	0 (0%)
Charity, Patients, General Public	0 (0%)	0 (0%)	0 (0%)	0 (0%)	0 (0%)	0 (0%)	0 (0%)
Other	0 (0%)	0 (0%)	0 (0%)	0 (0%)	0 (0%)	0 (0%)	0 (0%)

Supplementary Tabulated Data for Pye et al "**The use of quality checks and processes across digital histopathology: an initial survey from the Bigpicture consortium." Page 3/3**

	Pre Staining, N = 19	Staining, N = 19	Scanning, N = 19	Post Scan, N = 19	Digital Reporting and Display, N = 19	Reporting of Metadata, N = 19	Computational Analysis and AI, N = 19
**Opinion Q6). For digital histopathology how much does the variation introduced concern you?**							
A Great Deal	5 (31%)	6 (38%)	8 (50%)	4 (25%)	4 (25%)	4 (25%)	6 (38%)
A Little Bit	2 (12%)	5 (31%)	3 (19%)	5 (31%)	6 (38%)	6 (38%)	3 (19%)
I Am Not Sure	1 (6.2%)	0 (0%)	0 (0%)	1 (6.2%)	2 (12%)	1 (6.2%)	3 (19%)
Not Very Much	5 (31%)	4 (25%)	2 (12%)	3 (19%)	3 (19%)	2 (12%)	4 (25%)
I Don't Think It Is A Problem	3 (19%)	1 (6.2%)	3 (19%)	3 (19%)	1 (6.2%)	3 (19%)	0 (0%)
Opt Out	3	3	3	3	3	3	3
**Opinion Q7). How much do you believe image/data processing will be able to exclude the need for improved quality checks**							
Almost Entirely	2 (12%)	5 (31%)	7 (44%)	7 (44%)	6 (38%)	7 (44%)	9 (56%)
A Little Bit	4 (25%)	4 (25%)	2 (12%)	4 (25%)	5 (31%)	4 (25%)	3 (19%)
I Am Not Sure	1 (6.2%)	0 (0%)	0 (0%)	2 (12%)	3 (19%)	0 (0%)	0 (0%)
Not Very Much	7 (44%)	4 (25%)	6 (38%)	1 (6.2%)	2 (12%)	3 (19%)	3 (19%)
I Don't Think It Can	2 (12%)	3 (19%)	1 (6.2%)	2 (12%)	0 (0%)	2 (12%)	1 (6.2%)
Opt Out	3	3	3	3	3	3	3

	Q6 (n=16)	Q7 (n=16)
**Demographic**		
Clinical & Healthcare	6 (26%)	6 (26%)
University, Higher education, Academic Research	5 (22%)	5 (22%)
Pre-clinical, Non-Clinical, Pharmaceuticals	11 (48%)	11 (48%)
Computational, Health Data, SMEs, Other Companies	1 (4.3%)	1 (4.3%)
Biobank, 3rd Party Services, CRO	0 (0%)	0 (0%)
Public Regulator, Governmental Body, Learned Society or Funding Body	0 (0%)	0 (0%)
Charity, Patients, General Public	0 (0%)	0 (0%)
Other	0 (0%)	0 (0%)

QCC_SurveyOverview_Feb2023_v0.1

A COPY OF QUESTIONS CONTAINED IN THE ONLINE SURVEY

This document may be helpful to collate information from your colleagues whom may be more familiar with each of the seven stages if needed before submission.

Final submission should still be made online at the link below:

LINK TO ONLINE SURVEY FOR SUBMSSIONS : https://www.surveymonkey.co.uk/r/XQZ5FN3

This survey is for BIGPICTURE slide contributing centres to complete. It will help us understand what quality processes are already being used in the whole slide imaging (WSI) pathway. The survey will take under 10 minutes to complete.

The survey covers all stages of WSI production (see Fig1). You may not feel all stages are relevant to you / your center. There is an opt out question at the start of each stage, this will allow for submission of a survey for only the stages that are relevant to you / your center. Please answer everything you can.

This survey will be collected alongside your center reference which I will have provided to you on email, this is only to make sure we can chase centres not returning the data, and the key for this reference will only be available to limited named people at the BIGPICTURE quality co-ordination centre. Once the data collection period is over this data will undergo pseudonymisation and we will only share anonymized summary data.

Thank you for supporting this exciting and important piece of work for the Bigpicture quality co-ordination center. .

If you have any questions, please contact Hayley Pye at Hayley.pye@nhs.net

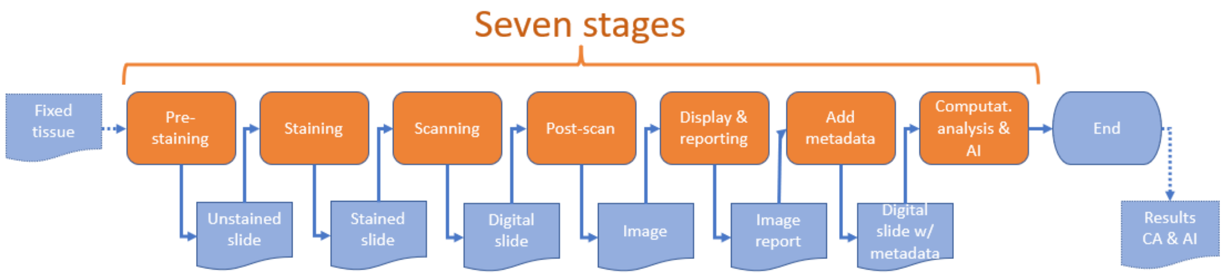

Fig1: Proposed workflow to produce a whole slide image.

<u>Workflow stages:</u>

1.Prestaining

•Includes: Tissue sample preparation, trimming, processing, embedding and sectioning.

2.Staining

•Includes: Tissue staining (any stain), cover slip mounting, slide inspection and storage.

3.Scanning

•Includes: Slide inspection and digitisation.

4.Post-scan

•Includes: Image inspection, preparation for image storage and upload to image management system.

5.Digital reporting and display

•Includes: Preparation of image for reporting, Image display to pathologist, display evaluation, report generation, (can also include clinical report validation where relevant).

6.Combine metadata and image

• Includes: extraction of data from pathology report and/or from sample production reports and its combination with the image (directly or in a linked format).

7.Computational analysis and AI

• Includes: Image displayed to user, preparation for computational analysis/AI

imi innovative medicines initiative
efpia

Instructions and demographics

Q1 Centre Reference

(This will have been provided over email)

Q1. Demographic data; sector.
(Please tick as many as you feel represent your sector):

☐ Clinical & Healthcare

☐ University, Higher education, Academic Research

☐ Pre-clinical, Non-Clinical, Pharmaceuticals

☐ Computational, Health Data, SMEs, Other Companies

☐ Biobank, 3rd Party Services, CRO

☐ Public Regulator, Governmental Body, Learned Society or Funding Body

☐ Charity, Patients, General Public

☐ Other

STAGE 1 (PRE STAINING)

1.Pre-staining

•Includes: Tissue sample preparation, trimming, processing, embedding and sectioning.

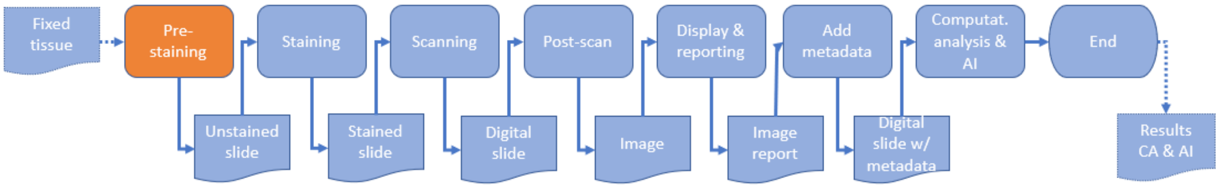

Q3. Are you able to answer questions on this stage of WSI production?
(if NO, this will bypass the questions and go to the next stage)

☐ NO, Our centre has absolutely no experience in this area.

☐ YES, I am happy to try and provide information for my centre in this area.

Q4. For the pre-staining stage of WSI production...

Quality checks and/or processes are carried out

☐ YES AT ALL STEPS ☐ YES, SOME STEPS ☐ NO ☐ UNSURE ☐ N/A

These have defined standards that need to be met (Internal or external)

☐ YES AT ALL STEPS ☐ YES, SOME STEPS ☐ NO ☐ UNSURE ☐ N/A

These are outlined in a managed document or QMS

☐ YES AT ALL STEPS ☐ YES, SOME STEPS ☐ NO ☐ UNSURE ☐ N/A

They are mandatory and need to be followed

☐ YES AT ALL STEPS ☐ YES, SOME STEPS ☐ NO ☐ UNSURE ☐ N/A

Records of the rate of compliance are kept

☐ YES AT ALL STEPS ☐ YES, SOME STEPS ☐ NO ☐ UNSURE ☐ N/A

imi innovative medicines initiative
efpia

STAGE 2 (STAINING)

2.Staining

•Includes: Tissue staining (any stain), cover slip mounting, slide inspection and storage.

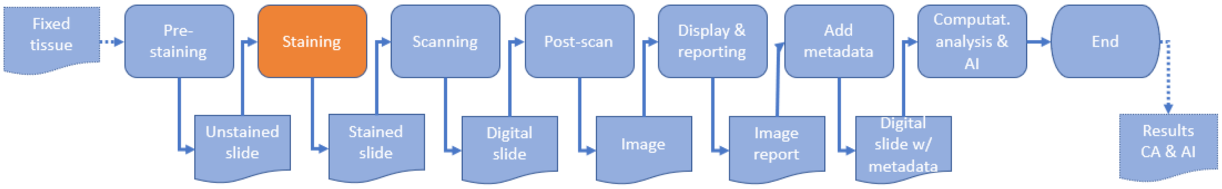

Q5. Are you able to answer questions on this stage of WSI production?
(if NO, this will bypass the questions and go to the next stage)

☐ NO, Our centre has absolutely no experience in this area.

☐ YES, I am happy to try and provide information for my centre in this area.

Q6. For the staining stage of WSI production...

Quality checks and/or processes are carried out

☐ YES AT ALL STEPS ☐ YES, SOME STEPS ☐ NO ☐ UNSURE ☐ N/A

These have defined standards that need to be met (Internal or external)

☐ YES AT ALL STEPS ☐ YES, SOME STEPS ☐ NO ☐ UNSURE ☐ N/A

These are outlined in a managed document or QMS

☐ YES AT ALL STEPS ☐ YES, SOME STEPS ☐ NO ☐ UNSURE ☐ N/A

They are mandatory and need to be followed

☐ YES AT ALL STEPS ☐ YES, SOME STEPS ☐ NO ☐ UNSURE ☐ N/A

Records of the rate of compliance are kept

☐ YES AT ALL STEPS ☐ YES, SOME STEPS ☐ NO ☐ UNSURE ☐ N/A

STAGE 3 (SCANNING)

3.Scanning

•Includes: Slide inspection and digitisation.

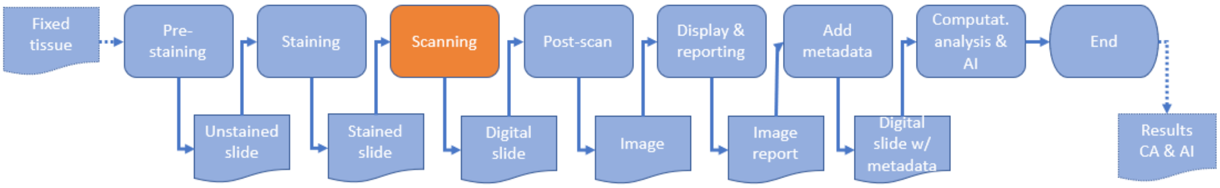

Q7. Are you able to answer questions on this stage of WSI production?
(if NO, this will bypass the questions and go to the next stage)

☐ NO, Our centre has absolutely no experience in this area.

☐ YES, I am happy to try and provide information for my centre in this area.

Q8. For the scanning stage of WSI production...

Quality checks and/or processes are carried out

☐ YES AT ALL STEPS ☐ YES, SOME STEPS ☐ NO ☐ UNSURE ☐ N/A

These have defined standards that need to be met (Internal or external)

☐ YES AT ALL STEPS ☐ YES, SOME STEPS ☐ NO ☐ UNSURE ☐ N/A

These are outlined in a managed document or QMS

☐ YES AT ALL STEPS ☐ YES, SOME STEPS ☐ NO ☐ UNSURE ☐ N/A

They are mandatory and need to be followed

☐ YES AT ALL STEPS ☐ YES, SOME STEPS ☐ NO ☐ UNSURE ☐ N/A

Records of the rate of compliance are kept

☐ YES AT ALL STEPS ☐ YES, SOME STEPS ☐ NO ☐ UNSURE ☐ N/A

imi innovative medicines initiative
efpia

STAGE 4 (POST SCAN)

4.Post-scan

•Includes: Image inspection, preparation for image storage and upload to image management system

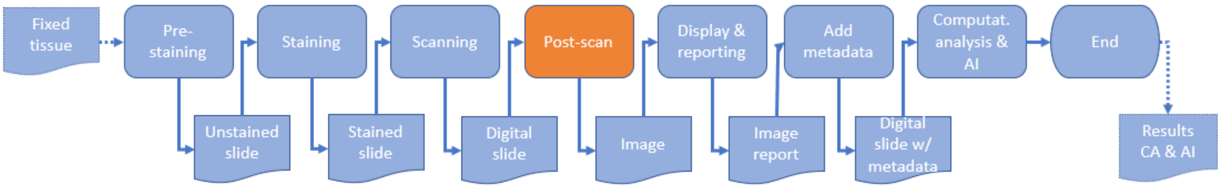

Q9. Are you able to answer questions on this stage of WSI production?
(if NO, this will bypass the questions and go to the next stage)

☐ NO, Our centre has absolutely no experience in this area.

☐ YES, I am happy to try and provide information for my centre in this area.

Q10. For the post-scan stage of WSI production...

Quality checks and/or processes are carried out

☐ YES AT ALL STEPS ☐ YES, SOME STEPS ☐ NO ☐ UNSURE ☐ N/A

These have defined standards that need to be met (Internal or external)

☐ YES AT ALL STEPS ☐ YES, SOME STEPS ☐ NO ☐ UNSURE ☐ N/A

These are outlined in a managed document or QMS

☐ YES AT ALL STEPS ☐ YES, SOME STEPS ☐ NO ☐ UNSURE ☐ N/A

They are mandatory and need to be followed

☐ YES AT ALL STEPS ☐ YES, SOME STEPS ☐ NO ☐ UNSURE ☐ N/A

Records of the rate of compliance are kept

☐ YES AT ALL STEPS ☐ YES, SOME STEPS ☐ NO ☐ UNSURE ☐ N/A

STAGE 5 (DIGITAL REPORTING AND DISPLAY)

5.Digital reporting and display

•Includes: Preparation of image for reporting, Image display to pathologist, display evaluation, report generation, (can also include clinical report validation where relevant).

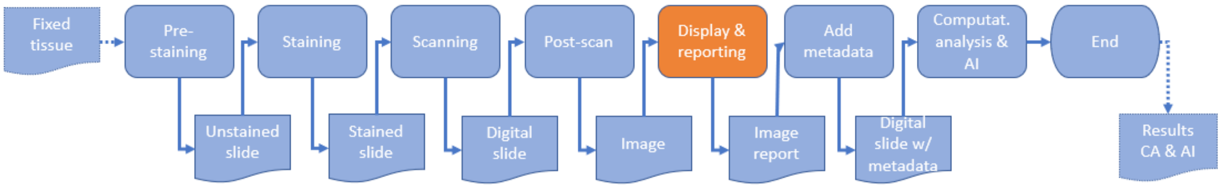

Q11. Are you able to answer questions on this stage of WSI production?
(if NO, this will bypass the questions and go to the next stage)

☐ NO, Our centre has absolutely no experience in this area.

☐ YES, I am happy to try and provide information for my centre in this area.

Q12. For the digital reporting and display stage of WSI production...

Quality checks and/or processes are carried out

☐ YES AT ALL STEPS ☐ YES, SOME STEPS ☐ NO ☐ UNSURE ☐ N/A

These have defined standards that need to be met (Internal or external)

☐ YES AT ALL STEPS ☐ YES, SOME STEPS ☐ NO ☐ UNSURE ☐ N/A

These are outlined in a managed document or QMS

☐ YES AT ALL STEPS ☐ YES, SOME STEPS ☐ NO ☐ UNSURE ☐ N/A

They are mandatory and need to be followed

☐ YES AT ALL STEPS ☐ YES, SOME STEPS ☐ NO ☐ UNSURE ☐ N/A

Records of the rate of compliance are kept

☐ YES AT ALL STEPS ☐ YES, SOME STEPS ☐ NO ☐ UNSURE ☐ N/A

innovative medicines initiative

STAGE 6 (REPORT METADATA)

6.Combine metadata and image

• Includes: extraction of data from pathology report and/or from sample production reports and its combination with the image (directly or in a linked format).

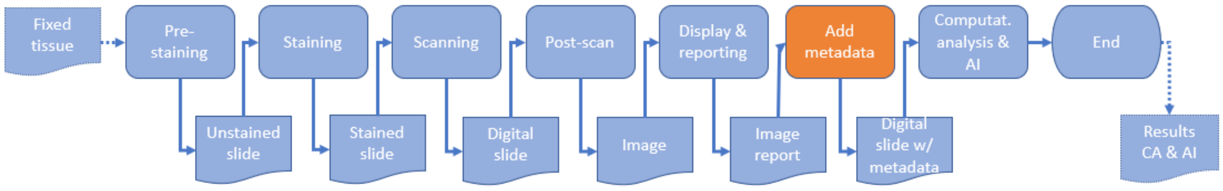

Q13. Are you able to answer questions on this stage of WSI production?
(if NO, this will bypass the questions and go to the next stage)

☐ NO, Our centre has absolutely no experience in this area.

☐ YES, I am happy to try and provide information for my centre in this area.

Q14. For the combine metadata and image stage of WSI production...

Quality checks and/or processes are carried out

☐ YES AT ALL STEPS ☐ YES, SOME STEPS ☐ NO ☐ UNSURE ☐ N/A

These have defined standards that need to be met (Internal or external)

☐ YES AT ALL STEPS ☐ YES, SOME STEPS ☐ NO ☐ UNSURE ☐ N/A

These are outlined in a managed document or QMS

☐ YES AT ALL STEPS ☐ YES, SOME STEPS ☐ NO ☐ UNSURE ☐ N/A

They are mandatory and need to be followed

☐ YES AT ALL STEPS ☐ YES, SOME STEPS ☐ NO ☐ UNSURE ☐ N/A

Records of the rate of compliance are kept

☐ YES AT ALL STEPS ☐ YES, SOME STEPS ☐ NO ☐ UNSURE ☐ N/A

STAGE 7 (COMPUTATIONAL ANALYSIS AND AI)

7.Computational analysis and AI

• Includes: Image displayed to user, preparation for computational analysis/AI

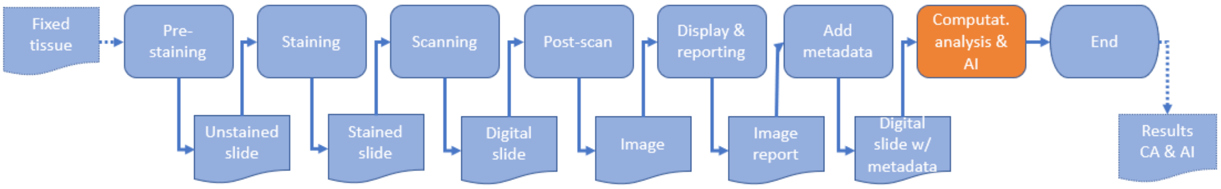

Q15. Are you able to answer questions on this stage of WSI production?
(if NO, this will bypass the questions and go to the next stage)

☐ NO, Our centre has absolutely no experience in this area.

☐ YES, I am happy to try and provide information for my centre in this area.

Q16. For the computational analysis and AI stage of WSI production...

Quality checks and/or processes are carried out

☐ YES AT ALL STEPS ☐ YES, SOME STEPS ☐ NO ☐ UNSURE ☐ N/A

These have defined standards that need to be met (Internal or external)

☐ YES AT ALL STEPS ☐ YES, SOME STEPS ☐ NO ☐ UNSURE ☐ N/A

These are outlined in a managed document or QMS

☐ YES AT ALL STEPS ☐ YES, SOME STEPS ☐ NO ☐ UNSURE ☐ N/A

They are mandatory and need to be followed

☐ YES AT ALL STEPS ☐ YES, SOME STEPS ☐ NO ☐ UNSURE ☐ N/A

Records of the rate of compliance are kept

☐ YES AT ALL STEPS ☐ YES, SOME STEPS ☐ NO ☐ UNSURE ☐ N/A

imi innovative medicines initiative
efpia

Opinion Questions

These two questions are for your current opinion and like the other questions the data will not be linked to your name or centre when presented.

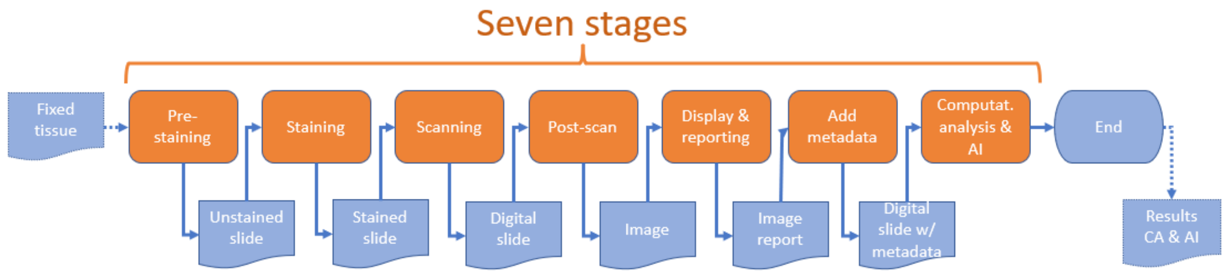

Workflow stages:

1.**Prestaining**
•Includes: Tissue sample preparation, trimming, processing, embedding and sectioning.

2.**Staining**
•Includes: Tissue staining (any stain), cover slip mounting, slide inspection and storage.

3.**Scanning**
•Includes: Slide inspection and digitisation.

4.**Post-scan**
•Includes: Image inspection, preparation for image storage and upload to image management system.

5.**Digital reporting and display**
•Includes: Image display to pathologist, preparation for reporting, report generation.

6.**Combine metadata and image**
• Includes: extraction of data from pathology report and/or from sample production reports and its combination with the image (directly or in a linked format).

7.**Computational analysis and AI**
• Includes: Image displayed to user, preparation for computational analysis/AI

Fig1: Proposed workflow to produce a whole slide image.

Q17. For digital histopathology how much does the variation introduced at each of the seven stages concern you?

Prestaining

☐ A great deal ☐ A little bit ☐ I am not sure ☐ Not very much ☐ I don't think it is a problem

Staining

☐ A great deal ☐ A little bit ☐ I am not sure ☐ Not very much ☐ I don't think it is a problem

Scanning

☐ A great deal ☐ A little bit ☐ I am not sure ☐ Not very much ☐ I don't think it is a problem

Post-scan

☐ A great deal ☐ A little bit ☐ I am not sure ☐ Not very much ☐ I don't think it is a problem

Digital reporting and display

☐ A great deal ☐ A little bit ☐ I am not sure ☐ Not very much ☐ I don't think it is a problem

Combine metadata and image

☐ A great deal ☐ A little bit ☐ I am not sure ☐ Not very much ☐ I don't think it is a problem

Computational analysis and AI

☐ A great deal ☐ A little bit ☐ I am not sure ☐ Not very much ☐ I don't think it is a problem

imi innovative medicines initiative
efpia

Q18.How much do you believe image/data processing will be able to exclude the need for improved quality checks at each of the sevens stages.

Prestaining

☐ Almost entirely ☐ A little bit ☐ I am not sure ☐ Not very much ☐ I don't think it can

Staining

☐ Almost entirely ☐ A little bit ☐ I am not sure ☐ Not very much ☐ I don't think it can

Scanning

☐ Almost entirely ☐ A little bit ☐ I am not sure ☐ Not very much ☐ I don't think it can

Post-scan

☐ Almost entirely ☐ A little bit ☐ I am not sure ☐ Not very much ☐ I don't think it can

Digital reporting and display

☐ Almost entirely ☐ A little bit ☐ I am not sure ☐ Not very much ☐ I don't think it can

Combine metadata and image

☐ Almost entirely ☐ A little bit ☐ I am not sure ☐ Not very much ☐ I don't think it can

Computational analysis and AI

☐ Almost entirely ☐ A little bit ☐ I am not sure ☐ Not very much ☐ I don't think it can

imi innovative medicines initiative
efpia

End

Thank you for supporting this exciting and important piece of work for the Bigpicture quality co-ordination center. We look forward to sharing the anonymised summary data with you in future.

All you need to do now is press 'submit data'

If you have any additional comment or feel like there were any key omissions in this survey please add them to the box below....

imi innovative medicines initiative
efpia
